# Comparison of Selected Metals Content in Cambodian Striped Snakehead Fish (*Channa striata*) Using Solar Drying System and Open Sun Drying

**DOI:** 10.1155/2015/470968

**Published:** 2015-01-22

**Authors:** Dayang Fredalina Basri, Nur Faizah Abu Bakar, Ahmad Fudholi, Mohd Hafidz Ruslan, Im Saroeun

**Affiliations:** ^1^School of Diagnostic & Applied Health Sciences, Faculty of Health Sciences, Universiti Kebangsaan Malaysia, Jalan Raja Muda Abdul Aziz, 50300 Kuala Lumpur, Malaysia; ^2^Solar Energy Research Institute (SERI), Universiti Kebangsaan Malaysia, 43600 Bangi, Selangor, Malaysia; ^3^Centre Kram Ngoy, No. 58, 318th Street, Sankat Tuol Svay Prey II, Olympic Quarter, Phnom Penh 12309, Cambodia

## Abstract

The content of 12 elements in Cambodian dried striped snakehead fish was determined using inductively coupled plasma mass spectrometry. The present study compares the level of the trace toxic metals and nutritional trace elements in the fish processed using solar drying system (SDS) and open sun drying (OSD). The skin of SDS fish has lower level of As, Pb, and Cd compared to the OSD sample. As such, the flesh of the fish accumulated higher amount of toxic metals during OSD compared to SDS. However, arsenic was detected in both samples within the safe limit. The nutritional elements (Fe, Mn, Mg, Se, Mo, Cu, Ni, and Cr) were higher in the skin sample SDS fish compared to OSD fish. These beneficial metals were not accumulated in the flesh sample SDS fish demonstrating lower level compared to drying under conventional system. The reddish coloration of the SDS fish was due to the presence of high Cu content in both the skin and flesh samples which possibly account for no mold formation 5 days after packaging. As conclusion, drying of Cambodian *C. striata* using solar-assisted system has proven higher content of the nutritious elements compared to using the conventional system despite only slight difference in the toxic metals level between the two systems.

## 1. Introduction

Cambodia's inland fisheries are among the world's largest and most diverse industry. While the country relies heavily on its natural resources and agricultural land to provide food and livelihoods for its rapidly growing population, the inland fisheries sector is perhaps the most valuable, which officially accounts for about 12% of gross domestic product (GDP) and provides most Cambodians with their key source of animal protein, calcium, and vitamin A [1].

The pollution of the aquatic environment with both essential and nonessential elements has attracted serious concern in the recent years because they are indestructible and most of them have toxic effects on organisms [2]. Heavy metals, particularly cadmium, lead, and arsenic constitute a significant potential health threat to human [3]. Fish is one of the sources of protein, vitamin, and mineral, and it contains essential nutrients required in human diet [4]. Fish has been widely accepted as a very important source of animal protein for supplementing both infants and adults diet [5, 6]. Fish drying is an age long practice of processing fish across the world to prolong its shelf-life and to conserve the quality [7].

Traditionally, fishes were dried under the open sun drying (OSD). OSD requires a large open space and depends on the availability of sunshine. It is a slow process and when drying of these animal products took a long time, bacterial spoilage during the slow operation occurred. Besides, OSD will not lower moisture content below about 15%, which is still too high for storage stability of food products. It also exposes products to birds, insects, and rodents and makes the products susceptible to contamination with foreign materials, such as dust and litter. The main contaminants that are likely to arise from bird droppings include fungus such as* Histoplasma capsulatum* and* Cryptococcus neoformans *[8] and bacteria* Chlamydophila psittaci *[9]. In addition, insects and fungi that thrive in moist conditions render the products unusable. In recent times, smoking kiln and solar drying system (SDS) are used to obtain product of high quality. SDS is a solar energy process that is well matched for drying of agricultural and fishery products in the tropical and subtropical countries [10]. According to [8], Phnom Penh receives an average of 5.3 hours of full sun every day with an average of about 2,490 hours of sunshine per year. The maximum fluctuation in solar radiation volume throughout the year is relatively low and has been estimated at 17%.

Many studies have reported on various methods of fish drying in tropical and subtropical countries. R. M. Davies and O. A. Davies [11] reported six different types of traditional fish processing techniques in Nigeria whereas an experimental study was carried out on solar tunnel dryer to dry fish [12]. A hybrid solar drying system with diesel burner was employed to dry salted silver jewfish in Johor, Malaysia [13, 14], and its drying characteristic was compared under open and solar drying [15]. The effect of traditional fish processing was investigated in terms of its nutritional value [16], proximate composition of raw and cooked Thai freshwater and marine fish [17], mineral composition, and proximate analysis of dried salted* Molva molva* L. and* Merluccius merluccius* L. [18]. Changes in nutritional and chemical composition of fried sardine (*Clupea pilchardus*) were reported [19] to be produced by microwave reheating and frozen storage.

It has been observed that different drying methods and processing have different effect on nutritional composition of fish [20]. The skin is compared to the flesh because the skin of* Channa striatus* has been known to possess toxic and lethal components [21] despite being edible among the local people. Bearing in mind that the quality of dried fish using different drying methods cannot be the same, as such, the objective of this study is to investigate the toxic and essential elements of striped snakehead fish (*Channa striata*) dried using OSD and SDS in Cambodia.

## 2. Material and Methods

### 2.1. Fish Sample

**Figure 1:**
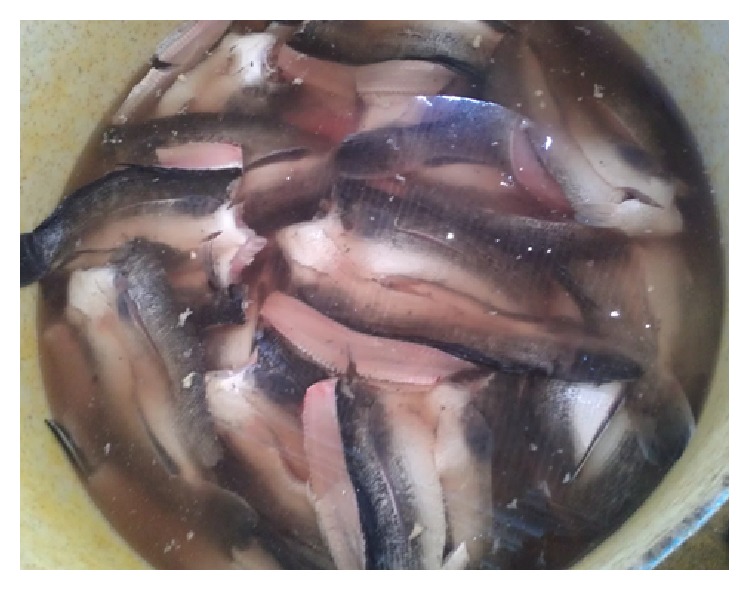
Salted striped snakehead.

Samples of the striped snakehead fish (*Channa striata*) as shown in Figure 1 were obtained from Cambodia. The mean length and weight of fresh fish were about 35 cm and 600 g, respectively. The striped snakehead fish was gutted and washed before cutting them prior to be subjected to two different methods of drying (OSD and SDS). The striped snakehead fish was soaked in separate containers that contained a 25% (w/v) brine solution of NaCl for 4 hr. A fish-to-brine ratio of 1 : 4 L was used [22, 23].

### 2.2. Solar Drying System (SDS)

**Figure 2:**
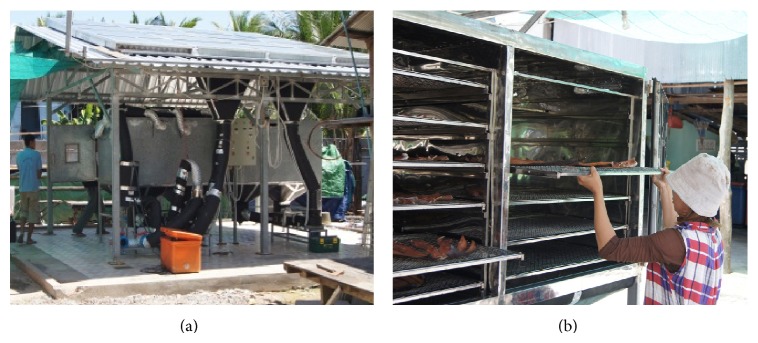
(a) Solar drying system (b) salted striped snakehead in solar drying chamber.

Solar drying system is shown in Figure 2(a), which comprised the forced-convection indirect type. The system consisted of a V-groove solar air collector, fans, electric heater, and drying chamber. The solar collector was of the back-pass V-groove which was connected in series. Setting the temperature at the required drying temperature can control the drying chamber temperature. Experiment was done between 9:00 a.m. and 5:00 p.m. corresponding to the sunshine duration in Cambodia. Drying experiment has been done on 62 kg striped snakehead. It was divided equally and then placed on 12 trays, as shown in Figure 2(b). During this process the drying temperature setting in drying chamber was fixed at 50°C and the flow rate was fixed at 0.07 kg/s. The data measured were air temperature (ambient temperature, air temperature inlet, and outlet of the collector), solar radiation, and air velocity, as well as the air temperature before it entered the dryer chamber, the temperature inside the dryer chamber, and the temperature of the air out of the dryer chamber. Air temperature was measured by T-type thermocouple, and the intensity of solar radiation was measured by pyranometer.

### 2.3. Open Sun Drying (OSD)

**Figure 3:**
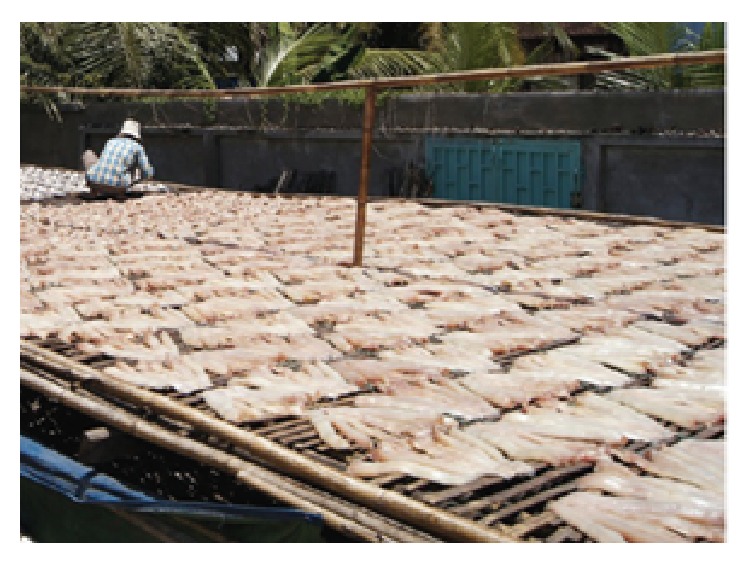
Open sun drying (OSD) method.

Simultaneously processed striped snakehead fish was dried in OSD as shown in Figure 3. The striped snakehead fish was placed on the bamboo tray in OSD from 9:00 a.m. to 5:00 p.m. At night, fishes were piled in plastic bins which were kept inside until the next morning and then continued to dry in the OSD. The striped snakehead fish was dried until the final moisture content reached about 40% w.b.

### 2.4. Inductively Coupled Plasma Mass Spectrometry (ICP-MS)

Samples were heated on the hot plate. The trace and minor elements content in samples were determined by using inductively coupled plasma mass spectrometry (ICP-MS ELAN 9000) (PerkinElmer, Sciex USA). The ICP-MS was set with the condition as stated in Table 1. For samples preparation, all the samples were processed using acid digestion method based on modified standard procedure [24]. Samples were rinsed using deionized water to get rid of all the contaminant, 5 g (wt/wt) of the samples was placed in the 50 mL beaker, and 10 mL of concentrated nitric acid was added. The mixture was heated on the hot plate until it changed to brown color. Sample was cooled and 10 mL of nitric acid was added. It was heated again until the solution once again begins to turn brown. The samples were heated again until the volume became 5 to 10 mL. After the mixture was evaporated to the desired volume, 2 mL of hydrogen peroxide solution was added. The process of adding hydrogen peroxide solution, heating, and cooling were repeated until the sample solution turned into clear sight. Next, samples were cooled and 2 mL of hydrochloric acid was added. Sample was heated again until the volume became 10 mL. Sample solution was diluted with deionized water until the volume eventually reached 100 mL. For standard solution, multielement stock solution Calibration number 3 from Perkin Elmer was used in this study. It contains 1000 mg/L of each element. Analytical calibration standards were prepared over the range 0–500 *μ*g/L for all tested elements.

## 3. Results and Discussion

The aim of the present study is merely to compare the level of the toxic and essential elements in the striped snakehead fish using OSD and SDS methods of drying instead of analyzing the quality of the fried fish product; hence, analysis of microbiology was not yet considered. Performance of SDS for striped snakehead fish is shown in Table 2.

In this study, the inductive coupled plasma mass spectrometry (ICP-MS) assay has been used to measure the elemental content in striped snakehead fish sample. Recoveries of trace metal contents in the present study are shown in Tables 3–5. A total number of 12 trace and minor elements (Pb, Cd, As, Zn, Mn, Cu, Cr, Mg, Mo, Fe, Ni, and Se) in the dried striped snakehead fish from Cambodia have been determined using ICP-MS after acid digestion. The concentration of 12 elements was determined based on their classification of toxic metals (As, Pb, and Cd) in Table 3 and nutritional trace elements (Fe, Mn, Mg, Se, Mo, Cu, Ni, Zn, and Cr) in Tables 4 and 5. The test was done in triplicates with the number of samples *n* = 3 for each analysis.

Table 3 shows the concentration of three heavy metals content in the skin and the flesh of the fish using two different drying methods. Out of these three toxic elements, As was detected at the highest concentration followed by Pb and Cd. As far as the skin samples are concerned, the fish subjected to OSD showed higher level of As, Pb, and Cd (8.72 ppb, 5.72 ppb, and 0.54 ppb) compared to the samples dried by SDS (7.32 ppb, 3.97 ppb, and 0.30 ppb). On the other hand, the flesh of the fish also accumulated high amount of As, Pb, and Cd (11.53 ppb, 3.87 ppb, and 0.31 ppb) during the process of OSD compared to when SDS technique (10.17 ppb, 1.43 ppb, and 0.18 ppb) was used. However, the levels of arsenic in both skin and flesh samples using both drying methods were well below the acceptable limit of 130 ppb for arsenic [25]. The acceptable limits for Pb and Cd are 240 ppb and 60 ppb, respectively [26].

As far as the skin samples are concerned, the nutritional trace elements were higher in the samples subjected to SDS with concentration of between 3.41 ppb and 2,019.69 ppb compared to OSD skin sample of within the range of 2.55 ppb to 1,550.55 ppb recorded for Se, Mn, Ni, Cu, Cr, Fe, Mo, and Mg as presented in Table 4. This means that the beneficial trace metals were conserved in the skin of the fried fish during the process of drying by SDS technique.

Generally, it was seen in Table 5 that SDS method of drying produced lower concentration of the beneficial trace elements (Mn, Se, Cr, Fe, Mo, and Mg) in the flesh of the fish samples compared to using conventional OSD method of drying the fish. The levels of Mn under SDS and OSD method were, respectively, 4.68 ppb and 4.73 ppb compared to the tolerable upper intake level (UL) for manganese in 70 kg adult at 11,000 *μ*g per day which corresponds to 157 ppb [27]. Although low level of manganese intake is necessary for human health, exposure to high manganese level has the potential to cause neurotoxicity [28]. As far as selenium is concerned, the SDS and OSD method displayed its concentration of 5.52 ppb and 5.62 ppb. According to the Institute of Medicine, the recommended dietary intake of selenium is 55 *μ*g per day for 70 kg adult equivalent to 0.80 ppb [29]. The Se levels in both samples are still considered within acceptable limit for consumption because the tolerable UL limit for selenium in 70 kg adult was set at 400 *μ*g per day which corresponds to 5.71 ppb [29]. In one study [30], selenium is reported to reduce vulnerability to mercury toxicity in humans and has protective effect for neonates against neurotoxicity from prenatal Mn exposure [31]. Chromium was detected at, respectively, 9.16 ppb and 10.67 ppb in SDS and OSD dried fish which is 20 times greater than the recommended average daily intake level (adequate intake) in 70 kg adult of 35 *μ*g per day for 70 kg adult equivalent to 0.50 ppb [32]. However, no adverse effects have been convincingly associated with excess intake of chromium from food or dietary supplements [27]. Besides, it has been found that some fish are capable of bioaccumulating Cr level nearly 100 times the concentration of Cr in the water [33]. The levels of Fe under SDS and OSD method were, respectively, 98.80 ppb and 130.71 ppb compared with the tolerable UL for iron in 70 kg adult was set at 45,000 *μ*g per day which corresponds to 642.86 ppb [27]. The levels of Mg under SDS and OSD method were, respectively, 3000.58 ppb and 3231.07 ppb compared with the tolerable UL value for magnesium in 70 kg adult was set at 350,000 *μ*g per day which corresponds to 5,000 ppb [27]. It can be deduced that the levels of Mn, Se, Cr, Fe, and Mg in the muscle of striped snakehead fish using both methods of drying were generally low when compared with the UL limit values. This is not the case for the level of molybdenum which recorded 239.04 ppb and 272.04 ppb in SDS and OSD samples. Logically, this is not considered safe for human consumption because it is almost 100 times the tolerable UL value for Mo in 70 kg adult as was set at 2,000 *μ*g per day or 28.57 ppb [34]. Nevertheless, the extremely high level of molybdenum in fishery products will not cause any harmful effects associated with high molybdenum level in human such as gout, anemia, and symptoms of copper deficiency. This is because of the rapid renal clearance of the majority of ingested molybdenum, which will likely prevent deleterious effects in the event of high intake [35].

**Figure 4:**
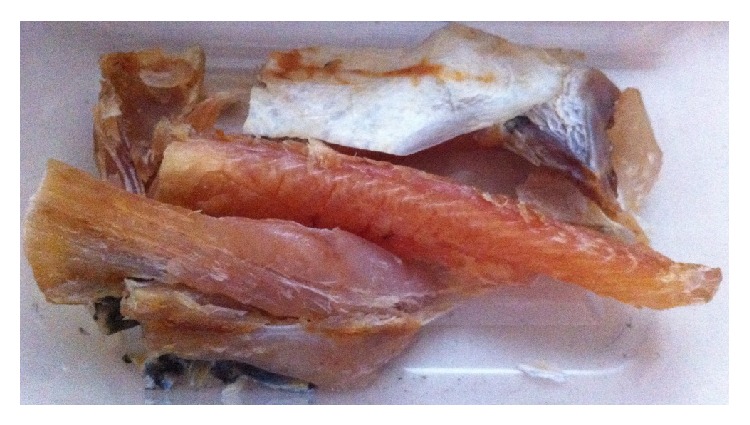
Quality of salted striped snakehead dried using SDS method: no formation of mold after day 5 packaging.

**Figure 5:**
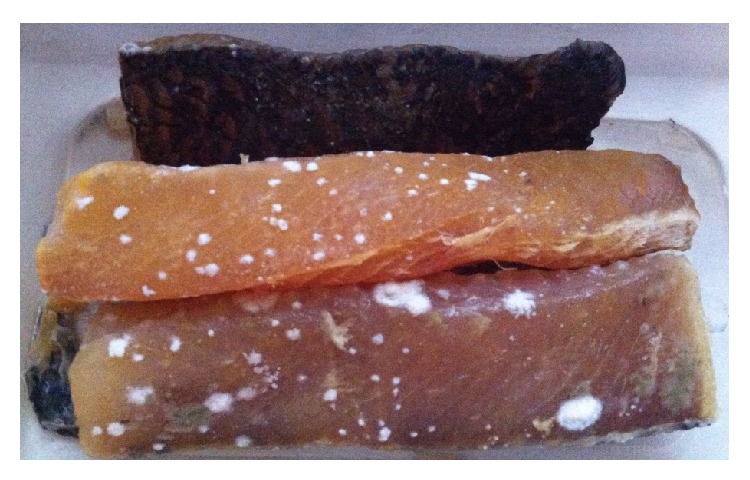
Quality salted striped snakehead dried using ODS method: formation of mold after day 5 packaging.

It is interesting to note that when SDS method was employed, the nutritious elements (Mn, Cr, Fe, and Mo) which were detected in high quantity in the skin of the fish samples (Table 4) were found to be in lower amount in the flesh of the same sample of the fish (Table 5). This is expected because when these important trace elements were greatly concentrated in the skin of the fish, they would not be accumulated in high concentration in the flesh of the same sample of dried fish. Ni and Cu however demonstrated higher level in both the skin and flesh of the fish samples under the technique of solar-assisted drying compared to OSD method. Therefore, this finding actually supported using of SDS method of drying the fish because the reddish coloration of the fishes dried under this condition is due to the presence of higher content of Cu in both the skin and flesh samples of solar-powered drying compared to both the skin and flesh samples of dried fishes subjected to OSD. In addition, the quality of fish was also preserved in the SDS method whereby no formation mold was observed after 5 days of packaging (Figure 4) compared to the presence of mold formation in OSD dried striped snakehead fish (Figure 5).

## 4. Conclusion

The finding from this study is important because it demonstrated that the different method of fish drying can influence their elemental contents and it is recommended that the drying method using SDS has proven to contain higher content of the nutritious trace elements compared to using the conventional open drying system. However, the level of toxic heavy metals only showed slight difference between the two systems. As conclusion, solar drying system is recommended for healthier eating and longer shelf-life of dried striped snakehead fish.

## Figures and Tables

**Table 1:** Operating conditions for ELAN 9000 ICP-mass spectrometer.

RF power	1000 W
Sampler diameter	1.1 mm
Sample skimmer cone	Ni
Nebulizer	Cross flow (Mainhard)
Peristaltic pump	1 mL/min
Argon flow rate plasma	15 L/min
Nebulizer flow	0.9 L/min
Spray chamber	Scott double pass

**Table 2:** Performance of SDS for salted striped snakehead.

Parameter and performance	Value	Unit
Initial weight of salted striped snakehead	60	kg
Final weight of salted striped snakehead	32	kg
Initial moisture content (wet basis)	80	%
Final moisture content (wet basis)	40	%
Air mass flow rate	0.07	kg/s
Average solar radiation	500	W/m^2^
Average ambient temperature	30	°C
Average drying chamber temperature	50	°C
Drying time	24	h
Solar energy	166	kWh
Heater and fans energy	100	kWh
The specific energy consumption	0.15	kg/kWh
Overall collector efficiency	31	%
Overall drying efficiency, up to 40% wb	10	%
Pick-up efficiency, up to 40% wb	94	%

**Table 3:** Concentration of toxic metals of salted striped snakehead.

Method of drying	Concentration of toxic metals in skin and flesh of dried fish (ppb)						Acceptable limit (ppb)		
	Skin			Flesh					
	As	Pb	Cd	As	Pb	Cd	As	Pb	Cd
SDS	7.321	3.973	0.304	10.171	1.427	0.180	* *130	* *240	* * 60
OSD	8.723	5.717	0.541	11.534	3.867	0.306			

**Table 4:** Concentration of nutritional trace elements in the skin of salted striped snakehead.

Method of drying	Concentration of nutritional trace elements in the skin of dried fish (ppb)								
	Fe	Mn	Mg	Se	Mo	Cu	Ni	Zn	Cr
SDS	241.970	5.591	2019.692	3.406	1064.478	9.854	8.634	136.236	25.858
OSD	216.327	4.423	1550.548	2.551	570.552	5.234	3.099	188.873	12.664

**Table 5:** Concentration of nutritional trace elements in the muscle of salted striped snakehead.

Method of drying	Concentration of nutritional trace elements in the flesh of dried fish (ppb)								
	Fe	Mn	Mg	Se	Mo	Cu	Ni	Zn	Cr
SDS	98.795	4.685	3000.576	5.515	239.035	6.383	1.859	146.649	9.159
OSD	130.710	4.729	3231.070	5.621	272.041	6.173	1.812	174.753	10.668
